# Transplantation and Replantation of Teeth

**Published:** 1876-11

**Authors:** William Taft


### DENTAL REGISTER.

VOL. XXX.] NOVEMBER, 1876. [NO. 11.

### TRANSPLANTATION AND REPLANTATION OF TEETH.

BY WILLIAM TAFT.

Though the general picture of civil history is often much deformed with records of the consequences of human misdeeds or abuses in power, yet the statesman and others concerned in the work of government, need such information in their efforts to avoid the dangers which have proved fatal to so many nations. In the history of science and of art, it is also necessary that attention should be directed, not merely to those successful steps by which valuable inventions and discoveries have been attained, but also to the errors and delusions which have so often proved so baneful to the growth of knowledge; in this way an acquaintance with the experience of others may be profitable, not only in teaching us to seek what is good, but also to avoid what is evil or pernicious. In the University of Glasgow, there is preserved the model of a machine intended to produce perpetual motion, by a clock maker, who spent seven years in this fruitless attempt to effect an impossibility. Had similar monuments of human folly been preserved in our museums, they might do much good in preventing the renewal of discarded systems and doctrines, while they also served a salutary check to the ardor of individuals whose love of change outstrips their ability to improve.

In applying remedies to the evils of the human system, and devising means to maintain its integrity, the errors of an in dividual are often attended with the most pernicious co sequences. More caution is necessary in taking steps which may entail death or protracted suffering on our fellow beings. And there is much to justify the care which the medical profession exercise over the introduction of innovations. In the profession of dentistry, also, there seems to be a necessity of like care in adopting certain modes of practice without due attention to the possible consequences with which they may be attended, or to the reasons which doomed them to be rejected in past times. Among these the method of transplanting and replanting teeth deserves attention, as they have been recently revived after a practical abandonment of nearly a century, particularly the former, which seems calculated to do more harm than might arise from sitting aside the more perfect modes of practice in vogue at the present day.

As the impression has prevailed among many in and out of the dental profession that the operation under consideration is of recent origin, while on the contrary, it almost antedates the art of dentistry itself, a history of it may prove interesting if not instructive, and also serve to show, that with all our boasted progress we are, in some respects, but little in advance of those of the last century.

The celebrated Ambroise Pare seems to be the earliest writer who alludes to the practice of inserting the natural teeth of one individual in the jaws of another. In a work written by this author about the close of the sixteenth century, he says: "I have heard it reported by a credible person, that he saw a lady of the prime nobility, who instead of a rotten tooth she drew, made a sound tooth, drawn from one of her waiting maids at the same time, to be substituted and inserted; which tooth, in process of time, as it were, taking root, grew so firm that she could chew upon it as well as upon any of the rest. I have this but by hearsay." It is probable that Pare himself was somewhat sceptical in regard to the matter, as he is particular to tell his readers that his evidence is only "hearsay."

Upon the subject of replanting of teeth, he gives his own experience, which furnishes us with the first authentic record of the operation. After referring to the diseases of the teeth, their treatment and care, the writer adds, "that if they (the teeth) become loose by a fall or blow, they must not be taken forth, but restored and fastened to the next that remain firm, but in time they will be confined in their sockets, as I tried in Antony de la Rue, who had his jaw broken with the pommel of a dagger, and three of his teeth were loosened and almost shaken out of their sockets; the jaw being restored, the teeth were also put in their places, and bound to the rest with a double waxed thread, for the rest I fed the patient with broths, jellies and the like, and I made astringent gargarisms of cypress nuts, myrtle berries, and a little alum boiled in oxycrate, and I wished him to hold it a good while in his mouth; by these means I brought it to pass that he, within a while after, could chew as easily upon these teeth, as upon the other." The edition from which these extracts were taken, was translated from the Latin, and compared with the French by Thomas Johnson, in 1617.

In the eighteenth century, when all kinds of knowledge were much advanced, and the merits of different systems were more fairly tested, the transplantation of teeth met with much disfavor from the most eminent representatives of the dental profession. This may be inferred from the following transcript from the work of Thomas Berdmore, the surgeon dentist to George the Third, published in 1668: "If," says the writer, "a tooth just extracted and instantly replaced in its socket, which it fits in a manner which no art can equal, fails of taking hold more frequently than it succeeds, and generally is attended with uneasiness and pain, if not with violent inflammation. What shall we say of those who pretend to supply one man with the teeth of another, with teeth which can not fit properly once in a thousand trials, that must necessarily press on the socket unequally, and therefore occasion inevitable pain and inflammation. The few instances in which they succeed, surely are not sufficient to counterbalance the hazard, and were people properly versed in the dentist's art they would certainly prefer the healing of the socket and the use of a well constructed artificial tooth or a human tooth with the root filed off, and formed to fit the void space exactly. For this will occasion none of the evils that attend the former practice, which is not only precarious, ineffectual and dangerous in general, but also immoderately expensive, for it is not to be supposed that any young person will sell a handsome tooth, to be torn out of his head, without being extremely well paid for the loss and pain."

This same author, upon the subject of replanting, says: "The surgeon's art has taught, that a tooth which has been partially or totally forced out of its socket, may be restored to its former situation and firmness, and may serve for use and ornament to the latest period of life." Then he proceeds to give directions for the performance of the operation, the conditions for success, etc. "In the case of a grown person," he says, "when the tooth is totally beat out, or when a surgeon is not at hand to reduce in the very instant, the swelling of the vessels, and extravasated blood prevent its being replaced as deep as before, and as a prominence above the rest of the teeth would expose it to future injury and pain, it is found necessary to cut off a little piece of the root, to smooth it well, to fill the hole in which the nerve formerly lodged with lead or gold; then to reduce it carefully and to fasten it o the neighboring tooth, etc."

In regard to the extracting teeth for the cure of diseased conditions, he continues: "A toothache often arises from a caries and disorder of the nerve; which last must be destroyed before any relief can be obtained. This, in the case before us, can be effected by extraction. The cariated part is then to be filed away, or, if the tooth be hollow, it is to be scraped clean, then filled with gold, lead, etc., and replaced as soon as possible."

It further appears that his faith in replantation, was not excessive, moreover that empiricism was abroad then as now, as may be inferred from the following:

"But after all that has been said on this subject, I think it necessary to add, for the sake of undissembled truth, the imputation of countenancing the impositions that occur every day that the success on all these occasions, however sufficient to justify the future trials and practice of honest and judicious people, is by no means equal to the extravagant assertions and promises of certain advertising imposters. In the most favorable circumstances it is more than an equal chance that a tooth once extracted or beat out will fasten again."

In a work published in 1774, by M. Patence, a dentist residing in London, in which he expresses in a quaint and rather medieval style, also his disapprobation of the unnatural system of transplantation: "I shall," he says, "next consider transplanting of teeth, which of late years has been practiced by several persons, and is no other than extracting the tooth of one person and placing it in the socket of another. It is generally taken from some poor person, who, not knowing the consequence attending such loss, consents to part with so useful an ornament, which can never be regained, for the sake of a few shillings. And it must certainly be highly offensive to the Almighty, for he never gave them for that purpose, neither are they their own to dispose of." In another passage the same writer points out some of the evils attending this course: "A case happened to a lady I lately had under my care, who had undergone the operation; the tooth transplanted was taken out of the mouth of a person that had the venereal disease, and was bound in with an

Indian weed; there presently issued an inflammation in both her upper and lower jaw bones; her face swelled to an enormous size, and was in the most curious pain; when she had been in this condition for some time, the tooth grew black, but had never fastened, and it was near twenty weeks before she got perfectly well."

It has of late been asserted by a member of the profession that by means of a process known only to himself, he could transplant teeth that had been extracted from the mouth any length of time, even after having carried them in his pockets until the roots were worn to a "polish." His statement aroused the incredulity of almost every one, but M. Patence oberves: "Transplantation may likewise be performed in another manner. Take a tooth from a skull, or one that has been drawn for some time, lay it in water for three months to soak, that it may open the pores; then take it out and put it in hot water for three hours, and when you have extracted the tooth from the person, this being fitted to the place, bind it fast with silk or weed to the other tooth, etc."

In a work published in Londen in 1778, by R. Wooffendale, a practical dentist, there is a more circumstantial detail of the mode in which teeth are to be transplanted, and of the uncertainties attending the undertaking. "The transplantation of teeth," says he, "is a very desirable operation when it succeeds, but it may not be improper to observe, that the success, in a great measure, depends upon chance. A dentist may know which tooth is proper for the purpose of being transplanted, as, whether it is a perfect one; whether the enameled part is of a proper size with regard to length, breadth, thickness, etc., but he can not know whether its root will correspond in shape and proportion with that of the one whose place it is to supply, till both are drawn, as it is well known the roots of teeth vary in these circumstances, notwithstanding the external and enameled parts perfectly accord." After some more lengthy details of the practice of transplanting and the circumstances which conspire to render it abortive, the same writer says: "By the above observations, I think it will appear that the success of this operation must depend on chance as much as on the art of the operator." In another passage he states: "There is an operation I have frequently performed with success, though not always, that may be mentioned in this place. It is when a tooth gives pain, and the nerve can not be readily destroyed, to take the tooth out, cut the decayed part away, stop the hollow with gold, and then replace it; if it is returned into its place without being stopped, and even becomes fast, it will decay as quick as if it had not been drawn, but by having this operation performed carefully, the tooth so replaced will often answer as well through life as if it had never been diseased."

The person with whom the transplantation of teeth found most favor, was John Hunter. This celebrated surgeon, whose writings on the teeth appeared at a somewhat later period than the authors from whom I have quoted, was so warm an advocate of dental transplantation, that he has been sometimes regarded as the author of the system. He endeavors to meet the difficulty of fitting transplanted teeth into new sockets, in consequence of the deviation of those organs from a common type and size. "Considering," says he, "the almost constant variety of the size and shape of the same class of teeth in different people, it would appear almost impossible to find the tooth of one person that should fit with any degree of exactness the socket of another; and this observation is supported, and indeed, would seem to be proved, by observing the teeth in skeletons. Yet we can actually transplant a tooth from one person to another, nature assisting the operation; it is done in such a way that she can assist. And the only way in which nature can assist, with respect to either size or shape, is by having the fang of the tooth rather smaller than the socket. The socket in this case grows to the tooth. If the fang is too large it is impossible indeed to insert it all in that state; however, if the fang should be originally too large, it may be made less, and this seems to answer the purpose as well.

"The success of the operation," continues the writer, "is founded on a disposition in all living substances to unite when brought into contact with one another, although they are of a different structure, and even the circulation is only carried on in one of them. Yet the hopes from the course of vital actions is lessened in the next sentence, in which he says: "This disposition to union is not so considerable in the more perfect or complex animals, such as quadrupeds, as it is in the more simple or imperfect; nor in old animals as in young, for the living principle in young animals and those of similar construction is not so much confined to or derived from one part of the body; so it continues longer in the part separated from their bodies, and would even appear to be generated in it for a time; while a part separated from an older or more perfect animal dies sooner, and would appear to have its life entirely dependent on the body from which it was taken."

In another part of his writings, he says of the transplanting of teeth: "Although this operation is in itself a matter of no difficulty, yet upon the whole, it is one of the nicest of all operations, and requires more chirurgical and physiological knowledge than any that comes under the care of the dentist. There are certain cautions necessary to be observed, especially if it be in the living tooth which is to be transplanted; because in that case it is meant to retain its life, and we have no great variety of choice. Much likewise depends on the patient; he should apply early and give the dentist all the time he thinks neccessary to get a sufficient number of teeth that appear to be of the proper size, etc."

From this it would appear that Hunter intended to meet the difficulty of a precise fit in the transplanted organs by causing one or more to submit to the pain and the loss of many teeth for the comfort or gratification of a single individual. Such an inhuman principle could be countenanced only in countries so rude and despotic as to recognize the most extreme differences between the upper and lower classes of society, and ignore entirely the natural rights of man. It is scarcely possible that such a system of self-mutilation for the benefit or the caprice of privileged classes, could hardly expect favor on this side of the Atlantic where customs and institutions are based on the great law of human freedom and equality. The system appears in a more odious light when we consider the small number of teeth for which transplantation can claim any reasonable hope of success. According to Hunter it succeeds best with the incisors, but in the case of the cuspidate and bicuspids, it is more uncertain; while all advocates of the system have pronounced it hopeless in the case of the grinding teeth.

Mr. Bell, editor of Hunter's Treatise on the teeth, in a foot note, says: "That the practice of transplanting originated with Hunter, under whose supervision it was frequently performed. Had the results of all these cases been known to him, it is probable his recommendation would not have been written. There is not, I believe, a single instance of its perfect success, and there are many in which it has been followed by even fatal results."

That the last named author was a strong advocate of replacing teeth after their having been removed through mistake, or forced from their socket by violence, is clearly shown from a further examination of his writings. "A tooth," he says, "beat out by violence, should be replaced as soon as possible. I would even recommend the experiment twenty-four hours after the accident, or as long as the socket will receive the tooth, which may be for some days,"

Hunter concludes his consideration of the subject of transplantation by giving a curious experiment in which he designs to show "that a living tooth when transplanted into some living part of an animal will retain its life, and the vessels of the animal shall communicate with the tooth. I took," he says, "a sound tooth from a person's head, then made a pretty deep wound with a lancet into the thick part of a cock's comb and pressed the fang of the tooth in to this wound and fastened it with threads passed through other parts of the comb. The cock was killed some months after, and I injected the head with a very minute injection; the comb was taken off and put into a weak acid, and the tooth being softened by this means, I slit the comb and tooth into two halves, in the long direction of the tooth. I found the vessels of the tooth well injected, and also observed that the external surface of the tooth adhered every where to the comb by vessels similar to the union of a tooth with the gums and sockets."

The circumstances which led to the abandonment of the system of transplanting after a fair trial by Hunter and many other able practitioners are clearly stated by Dr. Joseph Cox, an eminent surgeon dentist, in a work published in 1714, on the natural history of the teeth. In his words, "the ill success and unfortunate consequence that have sometimes occurred have caused the practice to be abandoned for many years past. The other methods of supplying the loss of teeth are so unexceptionable and invariably successful, that we have no reason to regret the failure of the method of transplanting. I might indeed have observed that this operation involved in it a defect of moral principle, as one person is injured and disfigured in order to contribute to the luxury or convenience of another." In detailing the case of a transplanted tooth which was worn by a gentleman for eleven years, he says: "It had never been quite fast, but during the past two or three years it had become quite loose; at length the crown broke off, and left the fang in the gum; on extracting it I found it to be absorbed all around in a most curious manner, and contrary to the common mode in which absorbents act upon the fangs of teeth, which is to begin at the point and extend upwards. In this way the absorbents, as if conscious that this tooth was an intruder, exerted their utmost power in order to eat it out rather than permit its continuance."

In a more recent work, J. Lefoule, a distinguished dentist of Paris, denounces transplanting in the following terms: "We consider it a sacred obligation to unite our voice to that of all the French dentists who have written upon the matter, in an unanimous cry of reprobation against men who do not blush to lend themselves to the selfishness of the rich, which would avail itself of the misery of the poor to extort a tooth, to replace one which perhaps was lost by intemperance and debauchery.

We repeat it that this traffic is almost banished from France and that there is not a single dentist here who would lend his aid to it. We regret that England and Germany do not yet follow so praisworthy an example."

It appears from contributions to the dental literature of the Germans that they are none the less severe in their denunciation of "Barbaric transplantation" than the author just quoted.

From all scientific records it appears that the practice of transplanting teeth was fairly tested in the past age and rejected both on account of the pernicious consequences with which it was attended and the introduction of other means for effecting the same end. The attempts to revive the exploded system at the present day are more unjustifiable, as great advancement has been made in the profession within the past fifty years, and greater resources have been brought within reach by the rapid progress in other arts and sciences. It would seem unnecessary to enter into further discussion respecting the inhumanity or impropriety of inflicting pain and injury on one individual in order to give another the chance of a very uncertain advantage. Nor is the evil confined to a mere failure of the operation or the troubles in the organs for which the remedies are designed. The danger of inocculation in the process of transplanting becomes the means of transferring the diseases of one individual into another and even the animal matter of the transplanted tooth may, under certain circumstances, prove very injurious by undergoing putrefactive change. In view of all these evils, uncertainties and dangers, we must discountenance the revival of a discarded system which could only have found favor in past times, when art, science and the morals of society were at a very low ebb, and which must be strongly condemned by the free and enlightened spirit of the present age.

